# Isolation and Molecular Analysis of Negeviruses in Mosquitoes (Diptera: Culicidae) from an Environmental Protection Area in the Brazilian Amazon

**DOI:** 10.3390/v18050501

**Published:** 2026-04-25

**Authors:** Bruna Alves Ramos, Daniel Damous Dias, Joaquim Pinto Nunes-Neto, José Wilson Rosa Junior, Durval Bertram Rodrigues Vieira, Valéria Lima Carvalho, Ana Lúcia Monteiro Wanzeller, Eliana Vieira Pinto da Silva, Maria Nazaré Oliveira Freitas, Landeson Junior Leopoldino Barros, Maissa Maia Santos, Jamilla Augusta de Souza Pantoja, Ercília de Jesus Gonçalves, Ana Claudia da Silva Ribeiro, Ana Cecília Ribeiro Cruz, Sandro Patroca Silva, Carine Fortes Aragão, Alexandre do Rosário Casseb, Livia Caricio Martins

**Affiliations:** 1 Department of Arbovirology and Hemorrhagic Fevers, Evandro Chagas Institute, BR 316, Km 07, s/n, Ananindeua 67030-000, Pará, Brazil; danieldias@iec.gov.br (D.D.D.); joaquimneto@iec.gov.br (J.P.N.-N.); josejr@iec.gov.br (J.W.R.J.); durvalvieira@iec.gov.br (D.B.R.V.); anawanzeller@iec.gov.br (A.L.M.W.); elianapinto@iec.gov.br (E.V.P.d.S.); landesonbarros@iec.gov.br (L.J.L.B.); maissasantos@iec.gov.br (M.M.S.); jamillapantoja@iec.gov.br (J.A.d.S.P.); erciliagoncalves@iec.gov.br (E.d.J.G.); anaribeiro@iec.gov.br (A.C.d.S.R.); anacecilia@iec.gov.br (A.C.R.C.); sandrosilva@iec.gov.br (S.P.S.); carinearagao@iec.gov.br (C.F.A.); liviamartins@iec.gov.br (L.C.M.); 2 Graduate Program in Virology, Evandro Chagas Institute, BR 316, Km 07, s/n, Ananindeua 67030-000, Pará, Brazil; maria_freitas17@hotmail.com; 3 Graduate Program in Parasitic Biology in the Amazon, State University of Pará, Perebebuí Bystreet, C Block, 2nd Floor, 2623, Belém 66095-662, Pará, Brazil; 4 Institute of Animal Health and Production, Federal Rural University of Amazônia, President Tancredo Neves Boulevard, 2501, Belém 66077-830, Pará, Brazil; alexcasseb@yahoo.com.br

**Keywords:** insect-specific viruses, virus diversity, Nelorpivirus, Sandewavirus

## Abstract

Mosquitoes are recognized as the arthropod group with the greatest vectorial capacity, and the viruses they transmit constitute a significant concern in the context of global One Health. In addition, these insects act as hosts for a wide diversity of insect-specific viruses (ISVs), which exclusively infect arthropods. Expanding knowledge of ISVs is particularly relevant, given their potential influence on arbovirus replication and their role in elucidating the evolutionary processes that shape virus–vector interactions. In this study, we report the isolation and molecular analysis of three negeviruses associated with different mosquito species of the genera *Culex*, *Coquillettidia*, *Mansonia*, and *Ochlerotatus*, collected in Belém, Pará State, in the Brazilian Amazon: Loreto virus, Wallerfield virus, and a putative new species, designated Terra firme virus. Eleven pools exhibited cellular alterations consistent with cytopathic effects in invertebrate C6/36 cells but showed no evidence of replication in vertebrate Vero cells. Notably, simultaneous infections by two or three negeviruses were detected in some mosquito pools, indicating the occurrence of multiple viral infections within individual samples. Genomic analyses revealed that the isolated strains share conserved domains with previously described isolates from other countries. Phylogenetic inferences demonstrated that the investigated strains are classified within the clades Nelorpivirus and Sandewavirus. Taken together, these findings expand the currently known diversity of the negevirus group and contribute to a more comprehensive understanding of its host range and geographic distribution.

## 1. Introduction

Insect-specific viruses (ISVs) constitute a group of viruses that naturally infect arthropods, particularly mosquitoes and sand flies. These viruses are capable of replicating in vitro in insect cells but lack the ability to infect or replicate in vertebrate cells, either in vitro or in vivo [1,2]. Initially, ISVs were thought to be restricted to the family *Flaviviridae*, a hypothesis supported by the isolation of flaviviruses such as *Cell-fusing agent virus* (CFAV, new name *Orthoflavivirus iunctionis*) [3], *Kamiti River virus* (KRV) [4], and *Culex flavivirus* (CxFV) [5]. However, recent advances in research, driven by the application of molecular approaches and high-throughput sequencing to characterize the virome of insects that are potential vectors of pathogens, have demonstrated that ISVs represent a genetically diverse group. These viruses are distributed across multiple viral taxa, including families that also comprise viruses capable of infecting vertebrates, invertebrates, and plants [1,2,6,7].

The negevirus group comprises insect-specific viruses known to produce in vitro infections with relatively high viral loads in arthropod cells [8,9,10]. It is currently divided into two major clades, Nelorpivirus and Sandewavirus [8,11,12,13], which include several recognized species such as Negev virus (NEGV), Loreto virus (LORV), Dezidougou virus (DEZV), Wallerfield virus (WALV), as well as unclassified negev-like viruses [2,9,12,13].

Representatives of the negevirus group correspond to spherical viral particles with diameters ranging from 45 to 55 nanometers [8,9]. Their genome consists of a non-segmented, positive-sense single-stranded RNA molecule, approximately 7039 to 10,054 nucleotides in length, organized into three coding regions (*open reading frames*—ORFs) and two non-coding regions [2,8,9]. From a functional perspective, these viruses harbor six protein domains that are essential for the replication cycle. ORF1 encodes the methyltransferase, helicase, and RNA-dependent RNA polymerase domains, whereas ORFs 2 and 3 encode, respectively, the DisA (or DisB) glycoprotein and the SP24 protein [2,8,9,14].

Reports of negevirus isolation from mosquitoes and sand flies collected in natural environments have been described in different regions of the world, including countries in Europe [10,15], Asia [16], Australia [17], and the Americas [7], highlighting the wide geographic distribution of this viral group. In Brazil, negev-like virus isolates have been reported in states from the Southeast, Central-West, and Northern regions, with confirmed occurrence in the Amazon region [9,13,14]. Within the Brazilian Amazon, strains of Negev virus (NEGV), Wallerfield virus (WALV), Piura virus (PIUV), Brejeira virus (BREV), and Cordoba virus (CDBV) have been detected naturally infecting mosquitoes collected in sylvatic environments under anthropogenic influence, particularly in areas adjacent to highways and mining zones in the state of Pará [9,14].

The conduct of studies focused on entomovirological surveillance and the molecular characterization of arboviruses and other arthropod-borne viruses has driven the discovery of new negeviruses in the Brazilian Amazon, expanding knowledge of the biological, ecological, and evolutionary characteristics of these viruses in the region [9,18,19]. In parallel, the advance of deforestation and unplanned urbanization represents a recurring environmental problem in the Amazon, requiring special attention to the emergence and re-emergence of viral diseases of relevance within the One Health, particularly arboviral infections [20,21]. Forest fragments embedded within large urban centers, such as those that comprise the Belém Metropolitan Region Environmental Protection Area (APA-Belém), harbor a high diversity of arthropods that are potential vectors of arboviruses and other infectious agents of medical importance. As such, these areas represent strategic settings for entomological and epidemiological surveillance efforts [22,23].

The Belém Metropolitan Region Environmental Protection Area (APA-Belém) is a conservation unit covering approximately 74.57 km^2^, located in the municipalities of Belém and Ananindeua, in the state of Pará, Brazil. Established in 1993, APA-Belém aims to protect the natural resources and historical heritage of the Belém metropolitan region. The area is composed of forest fragments of mixed ombrophilous vegetation, embedded within a predominantly urbanized matrix [24].

Owing to its insertion within a largely urban context, APA-Belém represents a strategic area for arbovirus surveillance studies. Investigations conducted since the 1950s, based on analyses of samples obtained from wild animals and hematophagous arthropods, have identified approximately 30 arbovirus species circulating in APA-Belém, highlighting the epidemiological relevance of the area for the maintenance and circulation of these viral agents [25,26,27,28,29].

Technological advances applied to diagnostic methods used in entomovirological surveillance studies, particularly the incorporation of molecular tools and bioinformatic analyses, have facilitated the identification of novel viruses and driven research on insect-specific viruses in APA-Belém [22].

Within this context, the present study aimed to report the isolation of viruses belonging to the negevirus group from pools of hematophagous arthropods of the family Culicidae collected in a forest remnant of the Amazon biome, as part of an arbovirus surveillance study conducted in APA-Belém between 2019 and 2020, as well as to describe the genomic characteristics of a putative novel negevirus, herein named Terra Firme virus (TERFV).

## 2. Materials and Methods

**Figure 1:**
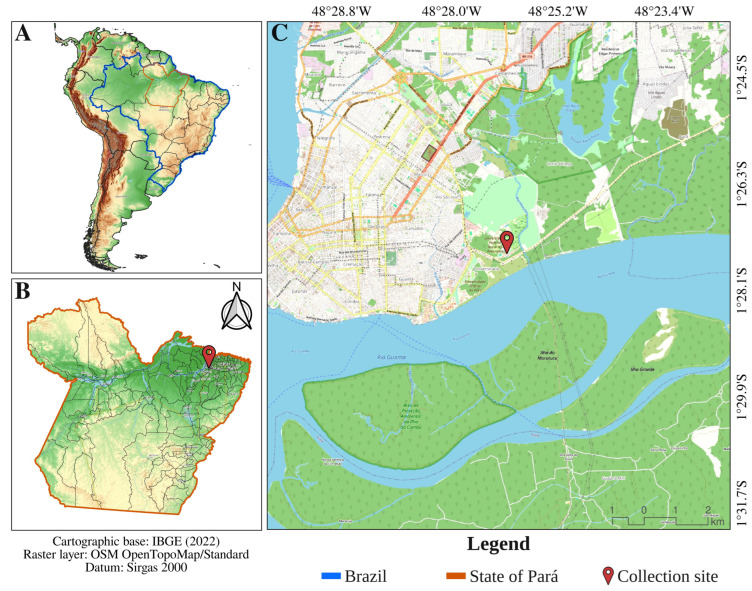
Location map of the mosquito collection site investigated in this study. The figure was produced using QGIS software v.3.22 (https://qgis.org/en/site/, accessed on 10 January 2026), with cartographic data from the Brazilian Institute of Geography and Statistics [33] and raster layers obtained from the OpenStreetMap project (https://www.openstreetmap.org/, accessed on 10 January 2026). Panel (**A**) shows South America, with Brazil highlighted in blue and the state of Pará in dark orange; panel (**B**) depicts the state of Pará with its administrative boundaries; and panel (**C**) indicates the secondary forest fragment within the Environmental Protection Area of the Metropolitan Region of Belém (Belém, Pará, Brazil), with the collection site marked by a red symbol.

The study was conducted at the main campus of the Federal Rural University of the Amazon (UFRA-Belém), located on Avenue Presidente Tancredo Neves, Terra Firme neighborhood, municipality of Belém, state of Pará, Brazil (geographic coordinates: 1°27′31″ S; 48°26′04.5″ W) (Figure 1). This research was conducted under authorization granted by the Brazilian Ministry of the Environment through the Biodiversity Authorization and Information System (SISBIO/IBAMA), under license number 63488. The sampling area lies within the jurisdiction of the Belém Metropolitan Region Environmental Protection Area (APA-Belém) and covers approximately 215.23 hectares. The site is characterized by the presence of discontinuous fragments of secondary vegetation, interspersed with semi-urban areas occupied by buildings, pastures, access roads, agricultural crops, and facilities dedicated to the rearing of domestic livestock. For the collection of hematophagous arthropods, four field expeditions were conducted on the UFRA-Belém campus, each lasting ten days.

The expeditions took place in February/March, June/July, and September/October 2019, corresponding respectively to the rainy, rainy–dry transition, and dry seasons. The fourth and final expedition was carried out in March 2020, representing the rainy season of that year.

Adult insects were collected using two distinct methods. To sample arthropods with predominantly diurnal activity, the protected human attraction technique (PHAT) was employed [30], using small entomological nets and oral aspirators. For the collection of individuals with crepuscular and nocturnal activity, CDC light traps (BioQuip Products^®^, Rancho Dominguez, CA, USA) were used, without additional attractants. Collections were carried out both at ground level and in the forest canopy. After collection, specimens were transferred to cryogenic tubes and immediately frozen in liquid nitrogen (−196 °C) and subsequently transported to the Department of Arbovirology and Hemorrhagic Fevers of the Evandro Chagas Institute (SEARB/IEC/SVSA/MS), where they were stored at −80 °C until taxonomic identification. At the Laboratory of Medical Entomology (SEARB/IEC), arthropods were sorted on a cold table (Eletrohospitalar, Brasília, Federal District, Brazil), maintained at approximately −38 °C, and identified based on external morphological characters, using the dichotomous key proposed by Forattini [31], with the aid of Zeiss Stemi 2000-C stereomicroscopes (Carl Zeiss, Göttingen, Germany). Genus and subgenus abbreviations followed the conventions proposed by Reinert [32]. Subsequently, identified specimens were grouped into pools containing one to 50 individuals of the same taxon. Each pool received a unique alphanumeric registration code and was stored at −80 °C until subsequent laboratory procedures.

### 2.1. Viral Isolation in Cell Cultures and Indirect Immunofluorescence Assay

For the viral isolation attempt, pools of mosquitoes were homogenized in 1 mL of Dulbecco’s phosphate-buffered saline (D-PBS 1×) (Life Technologies, Carlsbad, CA, USA), supplemented with 5% fetal bovine serum, 2% penicillin/streptomycin, and 1% amphotericin B (Gibco, Waltham, MA, USA), along with a 3 mm tungsten bead (Qiagen, Hilden, Germany). The pools were then macerated by agitation in a Tissuelyser II (Qiagen, Hilden, Germany) at 25 Hz for 1 min. The resulting suspensions were subjected to a freeze–thaw cycle at −80 °C for 24 h, followed by thawing and centrifugation at 10,000 rpm for 10 min at 4 °C in a refrigerated centrifuge. Subsequently, 150 µL of the clarified supernatant were simultaneously inoculated into individual tissue culture tubes (TPP^®^) containing monolayers of *Aedes albopictus* intestinal cells (clone C6/36; ATCC: CRL-1660) [34] and *Chlorocebus sabaeus* kidney cells (Vero; ATCC: CCL-81) [35].

Inoculated C6/36 cell cultures were maintained in modified Leibovitz medium with L-glutamine (L-15), supplemented with penicillin (10,000 U/L), streptomycin (10,000 µg/L), 1% non-essential amino acids, 2.95% tryptose phosphate broth, and 2% fetal bovine serum. In contrast, inoculated Vero cell cultures were maintained in medium 199, supplemented with penicillin (10,000 U/L), streptomycin (10,000 µg/L), and 2% fetal bovine serum (Gibco, Waltham, MA, USA). Cell cultures were maintained in a biological incubator for seven consecutive days, with C6/36 cultures incubated at 28 °C and Vero cultures at 37 °C under 5% CO_2_. During this period, inoculated cultures were examined daily under a Zeiss Axiovert S 100 inverted bright-field microscope (Carl Zeiss, Oberkochen, Baden-Württemberg, Germany) to detect cytopathic effect (CPE).

On the seventh day post-inoculation (7 dpi), all cell cultures inoculated with supernatants from homogenized hematophagous arthropods were subjected to indirect immunofluorescence assay (IFA) for screening of positive samples for arboviruses. Polyclonal antibodies specific to arbovirus groups of the families *Togaviridae* (genus *Alphavirus*), *Flaviviridae* (genus *Orthoflavivirus*), *Peribunyaviridae* (genus *Orthobunyavirus*), *Phenuiviridae* (genus *Phlebovirus*), and *Sedoreoviridae* (genus *Orbivirus*) (Supplementary Table S1) were used. These antibodies were produced *in-house* in Swiss albino mice (*Mus musculus*), diluted in PBS (1:20), following a protocol adapted from Gubler et al. [36].

For visualization of the immunoreaction, FITC-conjugated anti-mouse antibodies were employed at a dilution of 1:900 (Cappel, Solon, OH, USA), with the addition of Evans blue dye to facilitate the distinction between positive and negative cells. Slides containing the inoculated cell cultures were examined under an Olympus BX51 fluorescence microscope (Shinjuku, Tokyo, Japan) for the detection of fluorescent cells indicative of viral presence.

### 2.2. Viral RNA Extraction, Library Preparation, and Sequencing

Cell cultures inoculated with mosquito samples showing changes suggestive of cytopathic effect, together with defined results in the indirect immunofluorescence assay, were considered positive for viral presence and selected for sequencing. Supernatants from these cultures were collected for total RNA extraction and purification using the QIAamp Viral RNA^®^ kit (Qiagen, Hilden, Germany), according to the manufacturer’s instructions. The extracted RNA was used for first- and second-strand cDNA synthesis with the SuperScript™ VILO™ MasterMix Kit (Thermo Fisher Scientific, Waltham, MA, USA) and the NEBNext mRNA Second Strand Synthesis Module (New England Biolabs, Ipswich, MA, USA), respectively.

Subsequently, cDNA libraries were prepared following the protocol of the SureSelectQXT Whole Genome Library Prep Kit (Agilent Technologies, Santa Clara, CA, USA). Library quantification was performed using a Qubit^®^ 4.0 fluorometer (Life Technologies, Waltham, MA, USA), and library quality was assessed with an Agilent 2100 Bioanalyzer (Agilent Technologies, Santa Clara, CA, USA). Sequencing was carried out on the NextSeq 550 platform (Illumina, San Diego, CA, USA) in paired-end mode using the NextSeq 500/550 High Output Kit v2.5 (300 cycles – Illumina, San Diego, CA, USA).

### 2.3. Bioinformatics Analysis

#### 2.3.1. Quality Control

Raw sequencing data were initially subjected to quality control using FastQC [37] to assess read integrity and overall sequencing quality. Subsequently, read filtering and trimming were performed with Trim Galore v.0.6.7 [38], which was used to remove adapter sequences, reads shorter than 75 nucleotides, reads containing ambiguous bases, and bases with a Phred quality score below Q20. The resulting high-quality reads were then processed for ribosomal RNA depletion using SortMeRNA v.4.3.4 [39].

#### 2.3.2. Genome Assembly and Viral Taxonomic Classification

The processed reads were de novo assembled into contigs using the software Megahit v.1.2.9 [40] and SPAdes v.3.15.5 [41], applying, respectively, different sets of *k*-mer parameters in their default modes (21, 33, 55, and 77 for Megahit v.1.2.9; and 20, 40, 60, 80, and 100 for SPAdes). Subsequently, the generated contigs were subjected to taxonomic classification through alignments against the NCBI non-redundant protein database (nr), using the BLASTx mode of the DIAMOND v2.0.14 software [42], with the *e*-value threshold set to 1 × 10^−4^. The taxonomic classification results were then visualized and explored using Krona charts v.2.8 [43]. The identified viral contigs were subsequently searched in the NCBI-nr database and then imported into Geneious Prime v.2026.0.2 [44] for further analyses, including the prediction and extraction of open reading frames (ORFs) and the calculation of mean coverage using the Geneious mapper. Protein domain identification was performed using the InterProScan 5 v.77-108.0. [45].

As the Negevirus taxon has not yet been officially recognized by the International Committee on Taxonomy of Viruses (ICTV), operational criteria were adopted for species/strain demarcation, establishing a threshold of 85% amino acid sequence identity across the complete RNA-dependent RNA polymerase (RdRP). This criterion was defined based on previously published analyses [46,47,48], which suggest a threshold of approximately 80%, as well as on demarcation parameters commonly employed by the ICTV for insect viruses, typically ranging between 80% and 85% [46]. In addition, classifications were confirmed through analyses of phylogenetic relationships with previously described viral taxa (described below).

#### 2.3.3. Phylogenetic Analysis

The sequences of ORFs corresponding to RdRP were extracted and aligned with sequences of phylogenetically related viruses obtained from the NCBI database using multiple sequence alignment performed with Clustal Omega v.1.2.4 [49] in default mode. Subsequently, the alignments were visually inspected to remove ambiguous and poorly aligned regions. Phylogenetic reconstruction was carried out using FastTree v2.2 [50], employing the approximate maximum-likelihood method. For inferences based on amino acid sequences, the Jones–Taylor–Thornton substitution model with CAT approximation, which accounts for rate heterogeneity among sites, was applied (JTT + CAT). For analysis based on nucleotide sequences, the generalized time-reversible substitution model with CAT approximation was used (GTR + CAT). Branch support values were estimated using the Shimodaira–Hasegawa-like local likelihood test (SH-like), with 1000 internal site resamplings, as implemented in FastTree. The resulting topologies were visualized using FigTree v1.4.4 [51].

The nucleotide and amino acid identity matrices, obtained from multiple sequence alignments, were processed in the R environment (R Core Team, 2024) using the tidyverse package [52] for data handling and organization and pheatmap [53] for heatmap generation. Finally, figure editing and final adjustments were performed using Inkscape v1.4.2 (https://inkscape.org/, accessed on 6 November 2025).

## 3. Results

### 3.1. Viral Isolation and Indirect Immunofluorescence Assay

A total of 420 mosquito pools were inoculated into C6/36 and Vero cell cultures. Of these, 11 pools showed cellular alterations suggestive of cytopathic effect (CPE) in C6/36 cultures from the 4th day post-inoculation (dpi), observed during the fourth blind passage of the samples in cell culture (Table 1, Supplementary Figure S1).

In contrast, no alterations compatible with CPE were observed in Vero cell cultures throughout the seven consecutive days of incubation and monitoring. None of the pools that exhibited CPE-like alterations in C6/36 cells showed positive fluorescence reactions for the arbovirus groups tested by indirect immunofluorescence assay.

### 3.2. Nucleotide Sequencing and Viral Taxonomic Classification

Genome assembly resulted in 19 contigs, with lengths ranging from 8804 to 9150 nt. BLASTx analyses revealed the presence of three distinct negev-like viruses distributed among the mosquito samples, including two strains belonging to previously described viruses: Loreto virus (LORV) and Wallerfield virus (WALV), and one putative novel virus, provisionally named here Terra Firme virus (TERFV) in reference to the geographic origin of the analyzed specimens (Table 2).

**Figure 2:**
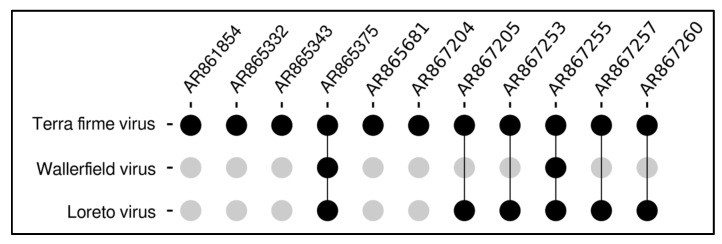
Distribution and co-occurrence of negeviruses in mosquito samples. Legend: The intersection plot illustrates the distribution of TERFV, WALV, and LORV across the analyzed samples. Black circles connected by vertical lines indicate the co-occurrence of multiple viral species within the same sample, whereas isolated black circles represent single infections. Gray circles represent the absence of the virus related to it.

Notably, simultaneous detections were identified within the same pools; samples AR865375 and AR867255 harbored all three viral species, whereas co-occurrence of LORV and TERFV was observed in samples AR867205, AR867253, AR867257, and AR867260 (Figure 2).

Despite the presence of multiple viral species within the same sample, the assembly algorithms successfully reconstructed individual, high-quality contigs for each virus. This allowed a detailed characterization of the viral sequences identified. Accordingly, six sequences were assigned to LORV, showing approximately 89.4% amino acid identity to the reference sequence (NC_034158), an average length of about 9140 nt, and coverage depths ranging from 20× to 9057×. TERFV accounted for the majority of detections, with 11 sequences whose lengths ranged from 8834 to 9065 nt and which exhibited approximately 65.2% amino acid identity to Kustavi negevirus (ON949944). In addition, two sequences were identified as WALV, both 8804 nt in length, derived from samples of *Ma*. (*Man.*) sp. and *Cx*. (*Mel*.) sp., showing 99.6% amino acid identity relative to WALV (KX518833) and coverage depths of 47× and 28×, respectively. All sequences yielded E-values of 0.0, confirming high statistical significance.

**Figure 3:**
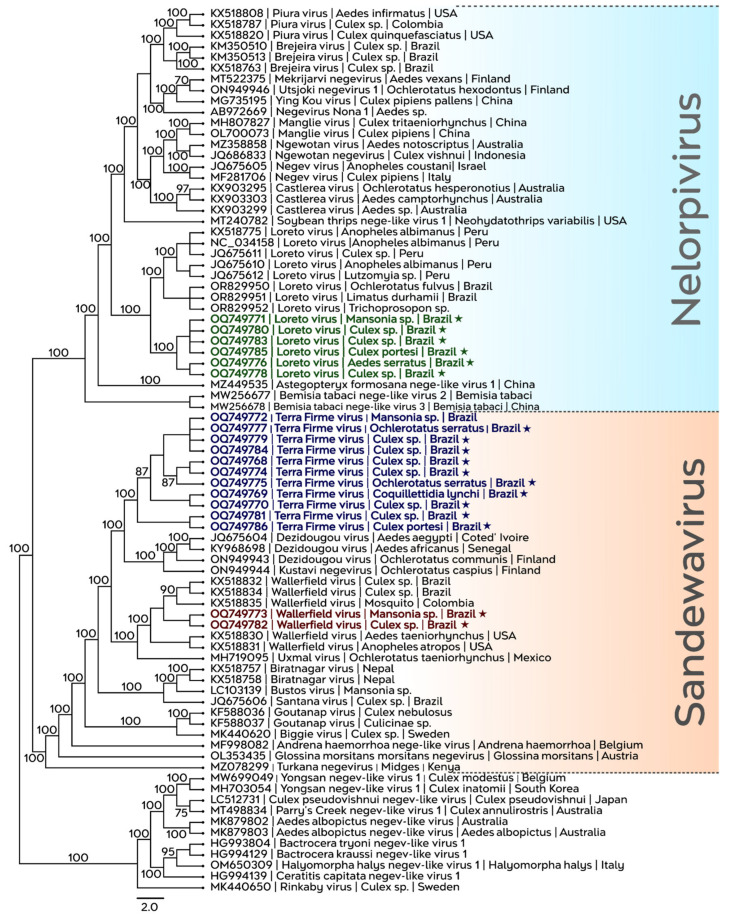
Approximate maximum-likelihood phylogenetic tree of viruses identified in this study. Colored clades indicate the Nelorpivirus (light blue) and Sandewavirus (orange) groups. Sequences generated here are highlighted and present a star: LORV (green), TERFV (dark blue), and WALV (red). The tree was midpoint-rooted, SH-like support values are shown at nodes, and GenBank accession numbers are provided next to virus names. The scale bar indicates amino acid substitutions per site.

To investigate the phylogenetic placement of the identified viruses, phylogenetic reconstructions were performed using the approximate maximum-likelihood method, based on RdRP sequences from the samples analyzed in this study, together with representative sequences of negev-like viruses publicly available (Figure 3). The analysis revealed that the 19 Brazilian sequences identified clustered into two distinct clades within the Negevirus taxon: Nelorpivirus and Sandewavirus. The sequences corresponding to the putative novel virus—Terra Firme virus (in dark blue), formed an exclusive and well-supported monophyletic clade (100% support), suggesting consistent evolutionary divergence in relation to other known sandewaviruses, including Dezidougou virus from Africa and Europe, and the Kustavi negevirus strain isolated in Finland. The sequences corresponding to LORV (in green) formed a monophyletic subgroup supported by high statistical support (100%), showing a basal relationship with sequences previously isolated in Brazil and Peru. WALV sequences from the APA-Belém also comprised a monophyletic clade supported by high statistical support, clustering with WALV sequences isolated in Brazil, Colombia, and the United States. Regarding the phylogenetic inference based on nucleotide sequences, similar topologies were observed (Supplementary Figure S2).

**Figure 4:**
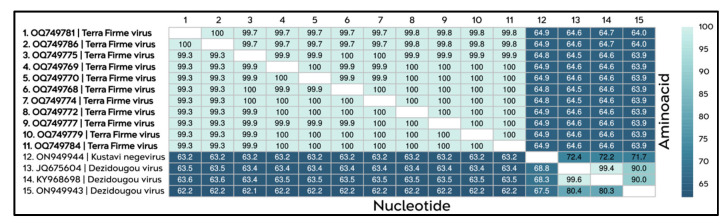
Identity matrix showing the percentages of nucleotide identity (**lower** diagonal) and amino acid identity (**upper** diagonal) among the TERFV sequences obtained in this study (highlighted in bold; sequences 1–11) and the sequences of Dezidougou virus and Kustavi negevirus (sequences 12–15). The color scale, represented by shades of blue, indicates identity percentages ranging from 65% to 100%, with lighter shades corresponding to higher levels of identity and darker shades indicating lower levels of identity among the sequences.

The identity matrix constructed from genomic sequences corresponding to ORF1, which encodes the RdRP of the TERFV strains isolated in this study, revealed a high degree of identity among them, with nucleotide and amino acid identity values ranging from 99% to 100%, respectively. In contrast, when compared with sequences of Dezidougou virus and Kustavi negevirus strains, the TERFV sequences exhibited substantially lower identity percentages, ranging from 63% to 65% at both the nucleotide and amino acid levels relative to the other negev-like viruses analyzed (Figure 4). These divergence levels fall well below the amino acid identity threshold previously established for species demarcation within the negevirus group (<85%), thereby providing strong support for the classification of TERFV as a distinct viral species.

**Figure 5:**
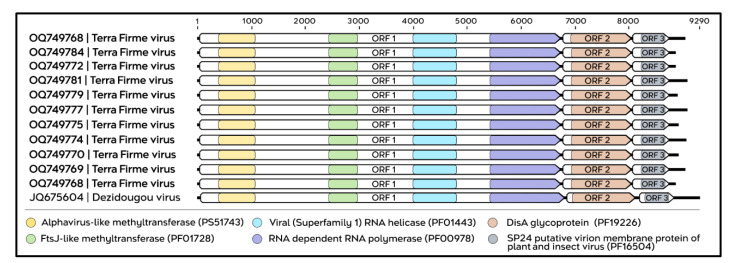
Genomic organization and functional domains of the TERFV sequences (highlighted in bold). The schematic illustrates the structural conservation among the analyzed sequences, highlighting the open reading frames (ORFs). Within ORF1, the conserved domains Alphavirus-like methyltransferase (PS51743, yellow), FtsJ-like methyltransferase (PF01728, green), viral RNA helicase (superfamily 1; PF01443, light blue), and RNA-dependent RNA polymerase (PF00978, purple) are shown. ORF2 encodes a DisA glycoprotein domain (PF19226, brown), whereas ORF3 encodes a putative membrane protein (putative virion membrane protein of plant and insect viruses; PF16504, gray).

The genomes of negev-like viruses identified in this study exhibit a structural architecture consistent with the pattern described for this viral group. The sequences corresponding to TERFV comprise positive-sense single-stranded RNA (ssRNA+) genomes, with lengths ranging from 8834 to 9065 nt. The genomic organization consists of three ORFs arranged in a collinear manner, flanked by a 5′ untranslated region (5′ UTR) of 45 nt and a variable 3′ untranslated region (3′ UTR) ranging from 90 to 321 nt (Supplementary Table S2), resulting in a coding region of approximately 8640 nt. The ORF1, located at the 5′ end of the genome, encodes a non-structural polyprotein containing conserved functional domains, including alphavirus-like and FtsJ-like methyltransferases, a viral helicase belonging to superfamily 1, and the RNA-dependent RNA polymerase (RdRp). This ORF is separated from ORF2 by a 35-nt intergenic region. The subsequent ORFs (ORF2 and ORF3), located at the 3′ end of the genome, encode putative structural proteins, identified as an envelope glycoprotein and a membrane-associated virion protein, respectively. In addition, TERFV shares the same set of conserved domains previously described for Dezidougou virus (Figure 5).

**Figure 6:**
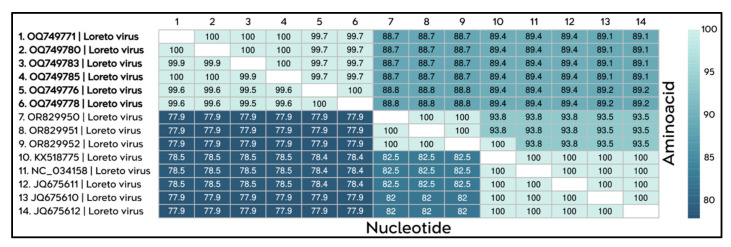
Identity matrix showing the percentages of nucleotide identity (**lower** diagonal) and amino acid identity (**upper** diagonal) among the LORV sequences obtained in this study (highlighted in bold; sequences 1–6) and publicly available LORV sequences (sequences 7–14). The color scale, represented by shades of blue, indicates identity values ranging from 80% to 100%, with lighter shades corresponding to higher levels of identity and darker shades indicating lower levels of identity among the sequences.

The identity matrix constructed from genomic sequences corresponding to ORF1 of LORV strains isolated in the present study (APA-Belém), together with ORF1 sequences from LORV strains previously isolated in Peru and Brazil (Figure 6), indicated that the isolates from APA-Belém exhibit high levels of identity among themselves, with nucleotide and amino acid identity values ranging from 99% to 100%. In contrast, when compared with Peruvian and Brazilian strains, the APA-Belém isolates showed high amino acid identity percentages (88–99%), accompanied by considerably lower nucleotide identity, ranging from 77% to 79%.

**Figure 7:**
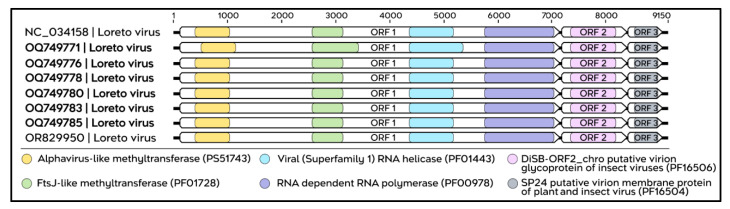
Genomic organization and functional domains of the complete LORV sequences isolated in this study. All analyzed genomes share the same set of conserved domains. ORF1 contains four conserved functional domains: Alphavirus-like methyltransferase (PS51743, yellow), FtsJ-like methyltransferase (PF01728, green), viral RNA helicase of superfamily 1 (PF01443, light blue), and RNA-dependent RNA polymerase (PF00978, purple). ORF2 encodes the DisB-ORF2_chro domain, corresponding to a putative insect virus virion glycoprotein (PF16506, pink), whereas ORF3 contains the SP24 domain, a putative virion membrane protein of plant and insect viruses (PF16504, gray).

In the present study, six complete sequences corresponding to the LORV genome were identified, with an approximate length of 9150 nt and a total coding region of 8889 nt (Supplementary Table S3). The genomic architecture observed is consistent with that previously described for this viral species, comprising three ORFs that share conserved functional domains with isolates previously reported in Brazil and Peru (Figure 7).

**Figure 8:**
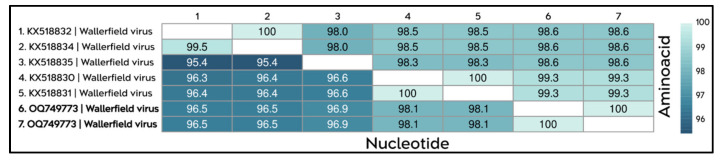
Identity matrix showing the percentages of nucleotide identity (**lower** diagonal) and amino acid identity (**upper** diagonal) among the WALV sequences obtained in this study (highlighted in bold; sequences 6–7) and publicly available WALV sequences (sequences 1–5). The color scale, represented by shades of blue, indicates identity values ranging from 96% to 100%, with lighter shades corresponding to higher levels of identity and darker shades indicating lower levels of identity among the sequences.

The identity matrix corresponding to the Wallerfield virus (WALV) sequences showed that the isolates from the APA-Belém share 100% identity among themselves at both the nucleotide and amino acid levels. When compared with WALV lineages previously described in Brazil and the United States, the local isolates exhibited high levels of conservation, with nucleotide identities ranging from 96.5% to 98.1% and amino acid identities ranging from 98.6% to 99.3% (Figure 8).

**Figure 9:**
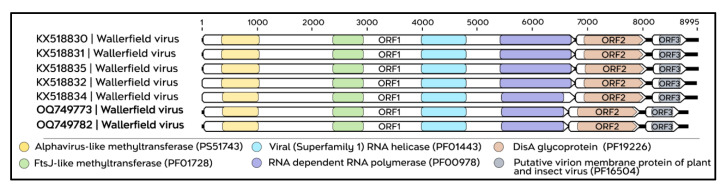
Genomic organization and functional domains of the complete WALV sequences isolated in this study. All analyzed genomes share the same set of conserved domains. ORF1 contains four conserved functional domains: Alphavirus-like methyltransferase (PS51743, yellow), FtsJ-like methyltransferase (PF01728, green), viral RNA helicase of superfamily 1 (PF01443, light blue), and RNA-dependent RNA polymerase (PF00978, purple). ORF2 encodes the DisA-ORF2_glycoprotein (PF19226, brown), whereas ORF3 contains the SP24 domain, a putative virion membrane protein of plant and insect viruses (PF16504, gray).

The complete genome sequences of WALV isolated in this study are approximately 9150 nt in length, with a total coding region of 8899 nt organized into three ORFs (Supplementary Table S4), which exhibit conserved functional domains when compared with isolates previously reported in Brazil (Figure 9).

## 4. Discussion

Overall, negeviruses comprise a group of insect-specific viruses (ISVs) known to cause natural infections in different arthropod species and to exhibit a wide geographic distribution [8,9,11]. Loreto virus (LORV) was first isolated in 1977 in the city of Iquitos, Peru, from pools containing mosquitoes of the genus *Culex* spp., and sand flies of the genus *Lutzomyia* [8,9,54]. In Brazil, the isolation of LORV was subsequently reported from pools of *Ochlerotatus fulvus*, *Trichoproposon digitatum*, and *Limatus durhamii* mosquitoes collected in Atlantic Forest fragments during entomovirological surveillance activities conducted in 2014 [54].

In the present study, LORV strains were isolated from pools containing *Mansonia* (*Man*.) sp., *Ochlerotatus serratus*, *Culex* (*Mel*.) sp., *Culex* (*Cux*.) sp., and *Culex* (*Mel*.) *portesi* mosquitoes, collected in the “Terra firme” forest fragment located within the Belém Environmental Protection Area (APA-Belém), in the Amazon biome. The isolated strains exhibited high amino acid sequence similarity but showed nucleotide divergences in comparison with LORV strains previously described in Peru and Brazil. These findings reinforce previous evidence suggesting that LORV can be classified as an insect-specific virus (ISV) with no strict host species specificity, infecting a wide diversity of dipterans. Moreover, the genetic variability observed among the isolates indicates that factors such as geographic origin and evolutionary time may exert a significant influence on the genetic diversification of this virus [8,54].

Isolates of Wallerfield virus (WALV) have been described from mosquito pools belonging to the genera *Culex*, *Aedes* Meigen, *Anopheles* Meigen, and *Deinocerites* Theobald in several countries, including the United States, Panama, Trinidad and Tobago, Colombia, and Brazil [7,9]. In the context of the Brazilian Amazon, the occurrence of WALV has been recorded in arbovirus surveillance studies conducted in areas subjected to intense environmental impact in the state of Pará, with the virus being isolated predominantly from mosquitoes of the genus *Culex* [9,14].

In the present study, a WALV strain genetically closely related to strains previously isolated in the state of Pará was obtained from pools containing *Culex* (*Mel*.) sp. and *Mansonia* (*Man*.) sp. mosquitoes, collected in a fragment of secondary forest located in an environment under recurrent anthropogenic influence. Notably, both pools were concomitantly infected with LORV and Terra Firme virus (TERFV). To date, there are no records in the literature of WALV infection in individuals of the genus *Mansonia*, as this virus has been traditionally associated with mosquitoes of the genus *Culex*. This finding expands the spectrum of potential hosts involved in the natural cycle of WALV.

In addition to LORV and WALV strains, a possible new virus to science, provisionally named Terra Firme virus (TERFV), was isolated from pools containing adult mosquitoes of the genera *Ochlerotatus* Lynch Arribálzaga, *Culex* Linnaeus, *Mansonia* Blanchard, and *Coquillettidia* Dyar. TERFV was able to induce cytopathic effect (CPE) in C6/36 cells (*Aedes albopictus*), without causing morphological alterations in the Vero cell monolayer. It was observed that C6/36 cells inoculated with supernatants from pools infected exclusively with TERFV exhibited moderate lytic CPE from the third passage onward, with generalized disorganization of the monolayer visible from the third day post-infection (dpi). In contrast, concomitantly inoculated Vero cells showed no apparent signs of infection. These findings suggest that TERFV presents replication restricted to insect cells, a characteristic compatible with ISVs. Previous studies have demonstrated that negeviruses are able to replicate efficiently in mosquito and sand fly cells, often reaching high viral titers in different insect species, but remain unable to infect laboratory animals or vertebrate-derived cell lines [8,9,11]. In the present study, it was not possible to evaluate TERFV replication in other vertebrate cell lines beyond Vero cells, which limits the complete definition of its in vitro susceptibility.

During the molecular analysis of the TERFV strains isolated in this study, it was determined that this virus possesses a genome composed of three open reading frames (ORFs), which harbor conserved protein domains commonly described in viruses of the negev-like group [8,9]. Genomic characterization revealed that TERFV shows a close genetic relationship with strains of Kustavi negevirus and Dezidougou virus, clustering within a phylogenetic subclade composed exclusively of TERFV sequences. This phylogenetic pattern supports the hypothesis that the isolates described herein correspond to a possible new negevirus.

In the present study, TERFV, LORV, and WALV strains were detected simultaneously infecting mosquitoes belonging to four distinct genera, collected from the same forest fragment. This finding suggests the existence of a common source of infection in the sampled environment, capable of sustaining the concurrent circulation of these viruses among different mosquito species. Previous evidence indicates that ISVs may constitute natural components of the microbiota of arthropods of the order Diptera, potentially competing for tissue binding sites and interfering with the transmission dynamics of insect-associated pathogens of medical importance [6].

Studies have demonstrated in vitro that negeviruses, such as Piura virus and Brejeira virus, are capable of reducing the viral load of vertebrate-pathogenic arboviruses of the genus *Orthoflavivirus* when inoculated into C6/36 cells. These findings suggest that ISVs may negatively interfere with the replication of certain arboviruses in coinfected vectors [55,56]. Despite the growing number of studies dedicated to the molecular characterization of new negeviruses isolated from mosquitoes across different continents and biomes, as well as investigations exploring their interactions with arboviruses in coinfected insect cells, understanding of the ecology of these ISVs remains incipient.

Studies conducted in Asia [57], Europe, and Oceania [17] have reported the isolation of new negeviruses from adult mosquitoes of the genera *Mansonia*, *Culex*, and *Aedes*, as well as from *Aedes* larvae collected in Japan in 2010 [58]. These findings suggest that these viruses may be transmitted among arthropods through both horizontal and vertical routes. The isolation of negeviruses from immature mosquito stages reinforces the hypothesis of vertical transmission from infected females to their offspring, most likely via the transovarial route, a mechanism currently considered the most plausible for the maintenance of these viruses in natural insect populations [8,58].

However, the genetic similarity observed between negeviruses and plant viruses, together with the wide diversity of insect species associated with infections by these viruses, suggests that additional mechanisms of horizontal dispersion may also occur in nature [9]. In this context, trophic interactions between insects and plants—through the consumption of sap or plant material containing viable viral particles—combined with environmental pressures and processes of mutation and genomic adaptation, may play a relevant role in the transmission and adaptation of negeviruses to mosquitoes.

In general, insects can act as vectors of plant viruses through two main mechanisms. The first, termed non-persistent transmission, involves a specific but reversible interaction between viral particles and the mouthparts of the vector. The second, known as persistent transmission, is characterized by viral infection followed by replication and dissemination in the intestinal epithelium, hemolymph, and salivary glands of the insect, rendering it permanently infected and capable of transmitting the virus to new hosts [59].

Previous studies have highlighted that negeviruses exhibit phylogenetic relationships with plant viruses belonging to the genera *Cilevirus*, *Higrevirus*, and *Blunervirus*, supporting the hypothesis of possible viral adaptation and horizontal transmission of these plant viruses to mosquitoes, potentially mediated by feeding behavior [2,9]. During the genomic analysis of the viruses isolated in the present study, it was observed that both viruses belonging to the Sandewavirus clade (WALV and TERFV) and those assigned to the Nelorpivirus clade (LORV) share conserved functional domains, including DisA, DisB, and SP24, with viruses infecting plants and insects, distributed among the families *Virgaviridae*, *Tombusviridae*, *Kitaviridae*, *Nodaviridae*, *Alphatetraviridae*, *Permutotetraviridae*, and *Iflaviridae* [1,2,60].

Analyses conducted by Kuchibhatla et al. [60] demonstrated that the DisA, DisB, and SP24 domains of negeviruses show significant homology with protein domains described in phytopathogenic viruses. According to the authors, this homology may have originated through horizontal RNA transfer events between distinct viruses during coinfection processes within the same host cell, in which genomic segments from one virus could be incorporated into the genome of another during virion assembly.

With regard to genomic organization, negeviruses generally exhibit three open reading frames (ORFs). The largest, ORF1, is responsible for encoding the RNA-dependent RNA polymerase. The smaller ORFs, ORF2 and ORF3—in which the functional domains DisA or DisB and SP24 are located—are associated, respectively, with the encoding of structural glycoproteins and envelope proteins. The occurrence of mutations in these regions is considered particularly relevant, as it may affect the expression and function of viral proteins involved in interactions with host cell receptors and, consequently, in viral adaptation processes [9,61]. Corroborating this hypothesis, a study conducted with a WALV strain isolated in Trinidad and Tobago in 2010 [7] described the presence of multiple glycosylation sites along the ORFs, which are potentially susceptible to recombination events. These findings reinforce the role of such elements in genetic diversification and evolutionary adaptation processes of negev-like viruses.

The Amazon harbors one of the greatest biological riches on the planet, being characterized by high faunal and floral diversity and by climatic conditions highly favorable to mosquito proliferation, which intensifies interactions among mosquitoes, plants, and a wide variety of vertebrate hosts [22,62]. It is widely recognized that these interactions among vectors, plants, and other animals play an important role in viral evolutionary and adaptive processes, particularly in the context of arboviruses [2]. In this context, it is plausible that the accumulation of mutations over time in specific genomic regions may have contributed—and may continue to contribute—to the emergence and diversification of negeviruses, potentially through viral transmission events involving plants and mosquitoes.

Environmental and ecological changes may influence interactions between mosquitoes and the assemblage of viruses that comprise their natural symbionts. Although some negeviruses exhibit relative genetic stability over time and across different geographic regions, pressures associated with climate change, urbanization, and environmental disturbances have the potential to alter vector population dynamics and behavior, with possible effects on the composition of the host virome. Such conditions may be associated with processes such as mutation, recombination, or viral adaptation, which, collectively, may contribute to variations in the host range observed among negeviruses [63,64,65].

Overall, the identification of new insect-specific viruses (ISVs) is of scientific relevance to viral ecology, considering their biological, ecological, and epidemiological importance within vector populations. In this context, continuous monitoring of the diversity and evolution of negeviruses across distinct ecological environments—through entomological surveillance actions combined with viral isolation and sequencing of mosquito viromes from areas under intense anthropogenic pressure—contributes to a better understanding of the role of these viruses in vector ecology. Moreover, such approaches provide a basis for assessing potential changes in their evolutionary patterns and host association dynamics.

## Figures and Tables

**Table 1:** Mosquito species and number of specimens per pool, sampling methods, and period of pools that showed CPE in C6/36 cells.

Sample ID	Species (Number per Pool)	Sampling	
		Method/Stratum	(Month/Year)
AR861854	*Culex* (*Mel.*) *spissipes* (9)	CDC/ground	Fev./2019
AR865332	*Coquillettidia* (*Rhy.*) *lynchi* (2)	CDC/ground	Fev./2019
AR865343	*Culex* (*Cux.*) sp. (32)	CDC/ground	Jul./2019
AR865375	*Mansonia* (*Man.*) sp. (12)	CDC/ground	Jun./2019
AR865681	*Culex* (*Mel.*) sp. (50)	CDC/ground	Out./2019
AR867204	*Ochlerotatus serratus* (26)	PHAT/ground	Mar./2020
AR867205	*Ochlerotatus serratus* (10)	PHAT/ground	Mar./2020
AR867253	*Culex* (*Cux.*) sp. (29)	CDC/ground	Mar./2020
AR867255	*Culex* (*Mel.*) sp. (25)	CDC/ground	Mar./2020
AR867257	*Culex* (*Mel.*) sp. (41)	CDC/ground	Mar./2020
AR867260	*Culex* (*Mel.*) *portesi* (31)	CDC/ground	Mar./2020

Legend: PHAT: protected human attraction technique.

**Table 2:** Genomic characterization and comparative analysis (BLASTx) of viral sequences identified in mosquito samples.

Virus Name (GenBank Accession)	Sample/Host	Novel/Strain	Length (nt)	Mean Coverage	Top BLASTx Hit Virus (Accession Number)	Amino Acid Identity (%)	QC (%)	E-Value
LORV (OQ749771)	AR865375/ *Ma* (*Man*) sp.	Strain	9133	142×	Loreto virus (NC_034158)	89.38	77	0.0
LORV (OQ749776)	AR867205/ *Oc. serratus*		9129	1441×		89.42		
LORV (OQ749778)	AR867253/ *Cx.* (*Cux*) sp.		9149	9057×		89.42		
LORV (OQ749780)	AR867255/ *Cx.* (*Mel*) sp.		9132	1083×		89.38		
LORV (OQ749783)	AR867257/ *Cx*. (*Mel*) sp.		9148	20×		89.38		
LORV (OQ749785)	AR867260/ *Cx. portesi*		9150	76×		89.38		
TERFV (OQ749768)	AR861854/ *Cx. spissipes*	Novel	8838	136,310×	Kustavi negevirus (ON949944)	65.20	76	0.0
TERFV (OQ749769)	AR865332/ *Cq. lynchi*		9026	3027×		65.25	74	
TERFV (OQ749770)	AR865343/ *Cx.* (*Cux*) sp.		8899	2459×		65.25	75	
TERFV (OQ749772)	AR865375/ *Ma*. (*Man*) sp.		8834	145×		65.25	76	
TERFV (OQ749774)	AR865681/ *Cx.* (*Mel*) sp.		9031	982×		65.16	74	
TERFV (OQ749775)	AR867204/ *Oc. serratus*		8901	4130×		65.25	75	
TERFV OQ749777)	AR867205/ *Oc. serratus*		9054	3543×		65.25	74	
TERFV (OQ749779)	AR867253/ *Cx.* (*Cux*) sp.		8885	1903×		65.25	75	
TERFV (OQ749781)	AR867255/ *Cx*. (*Mel*) sp.		9065	3433×		65.25	74	
TERFV (OQ749784)	AR867257/ *Cx*. (*Mel*) sp.		8834	111×		65.25	76	
TERFV (OQ749786)	AR867260/ *Cx. portesi*		9017	476×		65.25	74	
WALV (OQ749773)	AR865375/ *Ma.* (*Man*) sp.	Strain	8804	47×	Wallerfield virus (KX518833)	99.6	100	0.0
WALV (OQ749782)	AR867255/ *Cx*. (*Mel*) sp.		8804	28×		99.6	100	

Legend: Virus name (GenBank accession): Identified virus name followed by its GenBank accession number; Sample/Host: Sample code and mosquito species from which the virus was isolated. Novel/Strain: Classification as a previously known virus strain or a putative novel viral lineage/species. Length (nt): Total nucleotide sequence length. Mean coverage: Average sequencing depth across the genome. Top BLASTx hit virus: Closest viral match identified by BLASTx (with accession number). Amino acid identity (%): Percentage of amino acid identity relative to the BLASTx reference. QC (%): Query coverage, indicating the proportion of the sequence aligned to the reference. E-value: Statistical significance of the alignment.

## Data Availability

All data are contained within the article and Supplementary Materials. Additional data is kept by the corresponding authors and should be requested via email when needed.

## Supplementary Materials

The following supporting information can be downloaded at https://www.mdpi.com/article/10.3390/v18050501/s1, Figure S1: Monolayers of C6/36 cells inoculated with supernatants from mosquito pools were evaluated on the seventh day post-inoculation. (A) C6/36 cells inoculated with sample AR861854, showing cytopathic effect characterized by discrete monolayer disruption and formation of cell clumps. (B) C6/36 cells inoculated with sample AR865332, showing cytopathic effect with monolayer disruption and formation of cell clumps. (C) C6/36 cells inoculated with sample AR865343, showing cytopathic effect with monolayer disruption and formation of cell clumps. (D) C6/36 cells inoculated with sample AR865681, showing cytopathic effect with monolayer disruption and formation of cell clumps. (E) C6/36 cells inoculated with sample AR867204, presenting cytopathic effect with monolayer disruption, loss of cell format pattern and formation of cell clumps. (F) C6/36 cells inoculated with sample AR867205, showing cytopathic effect characterized by discrete monolayer disruption, increase in the size of some cells and formation of cell clumps. (G) C6/36 cells inoculated with sample AR867253, showing cytopathic effect with severe monolayer disruption and loss of cell format pattern. (H) C6/36 cells inoculated with sample AR867255, showing cytopathic effect with discrete monolayer disruption. (I) C6/36 cells inoculated with sample AR867257, showing cytopathic effect characterized by discrete monolayer disruption and formation of cell clumps. (J) C6/36 cells inoculated with sample AR867260, showing cytopathic effect with discrete monolayer disruption, loss of cell format pattern and formation of cell clumps. (L) C6/36 cells inoculated with sample AR865375, showing cytopathic effect with severe monolayer disruption formation of small cell clusters. (M) Uninoculated C6/36 cells (negative control). Scale bar: 150 µm. Magnification: 100×; Figure S2: Approximate maximum-likelihood phylogenetic tree of viruses identified in this study. Colored clades indicate the Nelorpivirus (light blue) and Sandewavirus (orange) groups. Sequences generated here are highlighted and marked with a star: LORV (green), TERFV (dark blue), and WALV (red). The tree was midpoint-rooted, SH-like support values are shown at nodes, and GenBank accession numbers are provided next to virus names. The scale bar indicates nucleotide substitutions per site; Table S1: Polyclonal antibodies used in the indirect immunofluorescence assay for the detection of arboviruses in C6/36 and Vero cell cultures inoculated with supernatants from macerated pools of the investigated mosquitoes; Table S2: Genomic organization of Terra Firme virus (TERFV) strains. Virus: Refers to the identified virus name. GenBank accession: Accession number of the deposited sequence. Genome length (nt): Genome size in nucleotides. ORF: Open Reading Frames. UTR: Untranslated regions. IR: Intergenic region; Table S3: Genomic organization of Loreto virus (LORV) strains. Virus: Identified virus name. GenBank accession: Accession number of the deposited sequence. Genome length (nt): Genome size in nucleotides. 5′-UTR (nt) and 3′-UTR (nt): Lengths of the untranslated regions. ORF1–ORF3 (nt/aa): Lengths of open reading frames in nucleotides and amino acids. IR1 and IR2 (nt): Lengths of intergenic regions; Table S4: Genomic organization of Wallerfield virus (WALV) strains. Virus: Identified virus name. GenBank accession: Accession number of the deposited sequence. Genome length (nt): Genome size in nucleotides. 5′-UTR (nt) and 3′-UTR (nt): Lengths of the untranslated regions. ORF1–ORF3 (nt/aa): Lengths of open reading frames in nucleotides and amino acids. IR1 and IR2 (nt): Lengths of intergenic regions.

## Abbreviations

The following abbreviations are used in this manuscript:

ISVs	Insect-Specific virus
CFVA	Cell-Fusing Agent virus
KRV	Kamiti River virus
CxFV	Culex flavivirus
NEGV	Negev virus
LORV	Loreto virus
DEZV	Dezidougou virus
WALV	Wallerfield virus
ORFs	Open Reading Frames
PIUV	Piura virus
BREV	Brejeira virus
CDBV	Cordoba virus
APA-Belém	Belém Metropolitan Region Environment Protection Area
TERFV	Terra Firme virus
UFRA	Federal Rural University of the Amazon
SISBIO/IBAMA	Biodiversity Authorization and Information System
IBGE	Institute of Geography and Statistics
PHAT	Protected Human Attraction Technique
SEARB/IEC	Department of Arbovirology and Hemorrhagic Fevers of the Evandro Chagas Institute
D-PBS	Dulbecco’s Phosphate-buffered saline
CPE	Cytopathic effect
IFA	Immunofluorescence Assay
dpi	Days post-inoculation
PBS	Phosphate-buffered Saline
ICTV	International Committee on Taxonomy of Viruses
RdRp	RNA-dependent RNA polymerase
