# Supplementary material for: Isolation and Molecular Analysis of Negeviruses in Mosquitoes (Diptera: Culicidae) from an Environmental Protection Area in the Brazilian Amazon
Source: Viruses. 2026 Apr 25;18(5):501. doi: 10.3390/v18050501 (PMC13211499; doi:10.3390/v18050501)
Supplement: Supplementary file 1 [file viruses-18-00501-s001.zip › viruses-4226272-supplementary.pdf]

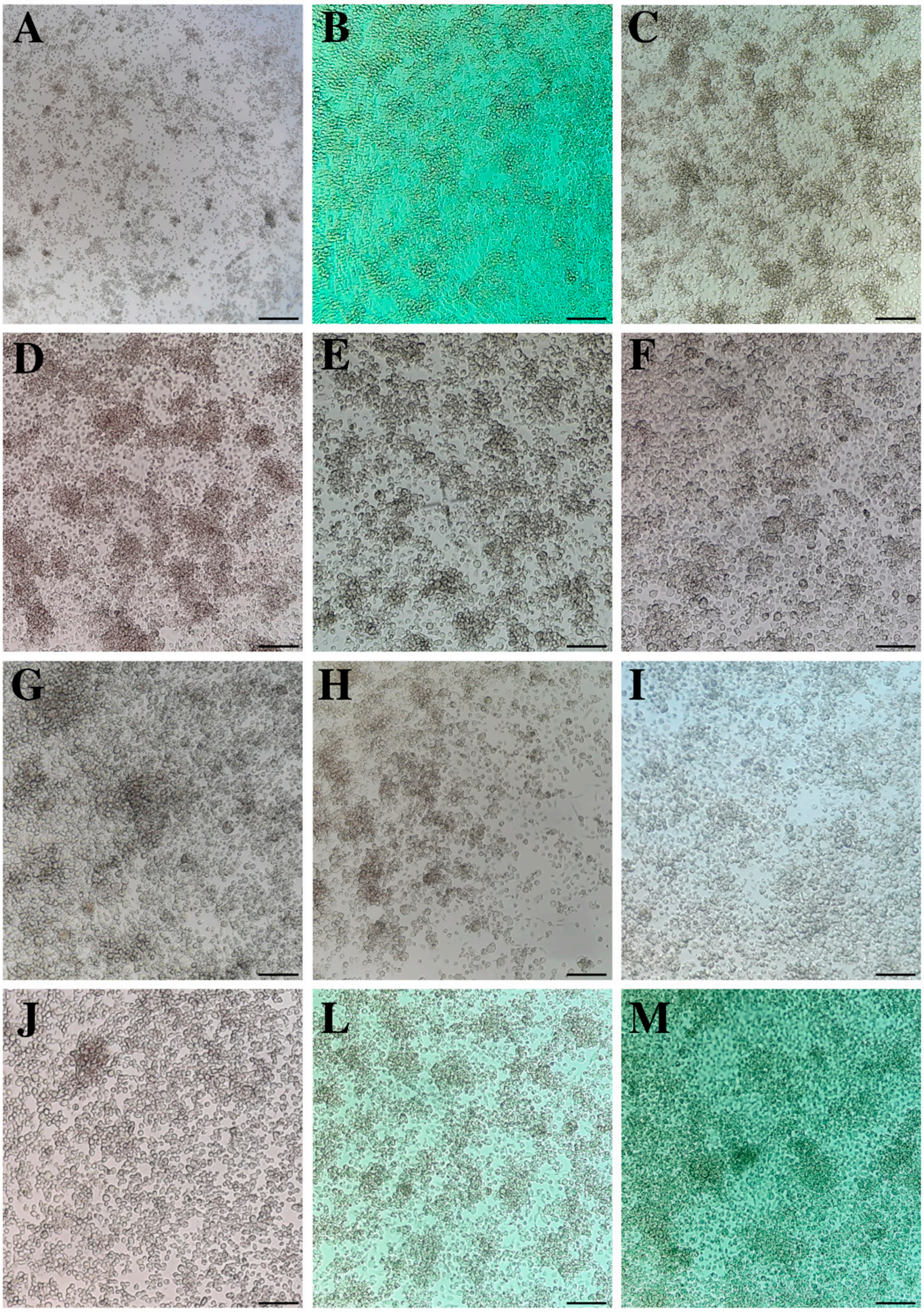

Figure S1. Monolayers of C6/36 cells inoculated with supernatants from mosquito pools were evaluated on the seventh day post-inoculation. (A) C6/36 cells inoculated with sample AR861854, showing cytopathic effect characterized by discrete monolayer disruption and formation of cell clumps. (B) C6/36 cells inoculated with sample AR865332, showing cytopathic effect with monolayer disruption and formation of cell clumps. (C) C6/36 cells inoculated with sample AR865343, showing cytopathic effect with monolayer disruption and formation of cell clumps. (D) C6/36 cells inoculated with sample AR865681, showing cytopathic effect with monolayer disruption and formation of cell clumps. (E) C6/36 cells inoculated with sample AR867204, presenting cytopathic effect with monolayer disruption, loss of cell format pattern and formation of cell clumps. (F) C6/36 cells inoculated with sample AR867205, showing cytopathic effect characterized by discrete monolayer disruption, increase in the size of some cells and formation of cell clumps. (G) C6/36 cells inoculated with sample AR867253, showing cytopathic effect with severe monolayer disruption and loss of cell format pattern. (H) C6/36 cells inoculated with sample AR867255, showing cytopathic effect with discrete monolayer disruption. (I) C6/36 cells inoculated with sample

AR867257, showing cytopathic effect characterized by discrete monolayer disruption and formation of cell clumps. (J) C6/36 cells inoculated with sample AR867260, showing cytopathic effect with discrete monolayer disruption, loss of cell format pattern and formation of cell clumps. (L) C6/36 cells inoculated with sample AR865375, showing cytopathic effect with severe monolayer disruption formation of small cell clusters. (M) Uninoculated C6/36 cells (negative control). Scale bar: 150 μm. Magnification: 100×.

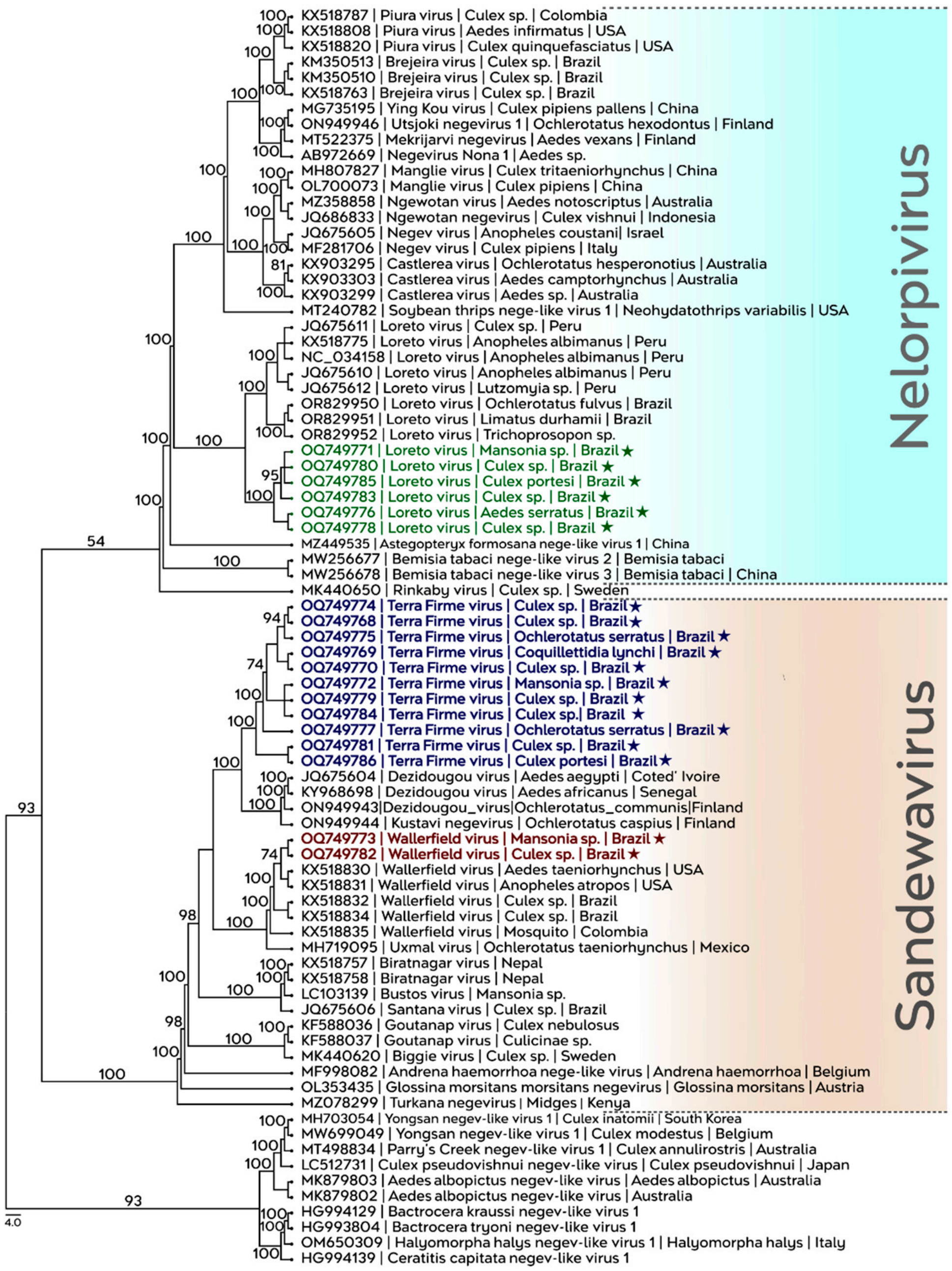

Figure S2: Approximate maximum-likelihood phylogenetic tree of viruses identified in this study. Colored clades indicate the Nelorpivirus (light blue) and Sandewavirus (orange) groups. Sequences generated here are highlighted and marked with a star: LORV (green), TERFV (dark blue), and WALV (red). The tree was midpoint-rooted, SH-like support values are shown at nodes, and GenBank accession numbers are provided next to virus names. The scale bar indicates nucleotide substitutions per site.

Supplementary Table S1. Polyclonal antibodies used in the indirect immunofluorescence assay for the detection of arboviruses in C6/36 and Vero cell cultures inoculated with supernatants from macerated pools of the investigated mosquitoes.

Genus	Antigenic group	Viruses
Alphavirus	A	*Alphavirus aura, Alphavirus eastern, Alphavirus mayaro, Alphavirus mucambo, Alphavirus pixuna, Alphavirus una, Alphavirus western, Alphavirus chikungunya, Alphavirus trocara*
Orthoflavivirus	B	*Orthoflavivirus denguei* (1-4), *Orthoflavivirus flavi, Bussuquara virus, Orthoflavivirus cacipacoreense, Orthoflavivirus ilheusenses, Orthoflavivirus nilense, Orthoflavivirus louisense, Orthoflavivirus zikaense.*
Orthobunyavirus	Guamá	*Orthobunyavirus ananindeuaense, Bimiti orthobunyavirus, Orthobunyavirus catuense, Orthobunyavirus guamaense, Orthobunyavirus mirimense, Moju orthobunyavirus, Orthobunyavirus timboteuaense.*
	Capim	*Orthobunyavirus acaraense, Benevides orthobunyavirus, Orthobunyavirus benficaense, Orthobunyavirus capimense, Orthobunyavirus guajaraense, Orthobunyavirus bushbushense, Moriche orthobunyavirus*.
	Bunyamwera	*Orthobunyavirus iacoense, Kairi orthobunyavirus, Orthobunyavirus macauaense, Orthobunyavirus maguariense, Sorocaba orthobunyavirus, Tucunduba orthobunyavirus, Taiassui orthobunyavirus, Xingu orthobunyavirus.*
	Simbu	*Orthobunyavirus oropoucheense*
Phlebovirus	Phlebotomus	*Phlebovirus alenquerense, Phlebovirus ambeense, Ariquemes phlebovirus, Belterra phlebovirus, Phlebovirus bujaruense, Phlebovirus candiruense, Phlebovirus icoaraciense, Joá phlebovirus, Phlebovirus itaporanguense, Jacundá phlebovirus, Morumbi phlebovirus, Mucura phlebovirus, Phlebovirus mugumbaense, Phlebovirus oriximinaense, Pacuí phlebovirus, Phlebovirus saloboense, Phlebovirus taparaense, Phlebovirus turunaense, Phlebovirus uriuranaense, Phlebovirus urucuriense.*
Orbivirus	Changuinola	*Acatinga virus, Acurené virus, Almeirim virus, Altamira virus, Anapú virus, Araçaí virus, Aratau virus, Aruana virus, Arawetê virus, Assurinis virus, Bacajaí virus, Bacuri virus, Balbina virus, Barcarena virus, Breves virus, Canindé virus, Canoal virus, Catetê virus, Orbivirus changuinolaense, Coari virus, Gorotire virus, Gurupi virus, Iopaka virus, Ipixaia virus, Irituia virus, Iruana virus, Itaboca virus, Jamanxi virus, Jandaia virus, Jari virus, Jatuarana virus, Jutaí virus, Kararaô virus, Melgaço virus, Monte Dourado virus, Ourém virus, Pacajá virus, Parakanã virus, Poranati virus, Parauapebas virus, Parú virus, Pependana virus, Pindobai virus, Piratuba virus, Purus virus, Rio Mutapi virus, Saracá virus, Serra Sul virus, Surubim virus, Tapiropé virus, Tekupeú virus, Timbozal virus, Tocantins virus, Tocaxá virus, Tuerê virus, Tumucumaque virus, Uatamã virus, Uxituba virus, Xaraíra virus, Xiwanga virus.*

Supplementary Table S2. Genomic organization of Terra Firme virus (TERFV) strains. Virus: Refers to the identified virus name. GenBank accession: Accession number of the deposited sequence. Genome length (nt): Genome size in nucleotides. ORF: Open Reading Frames. UTR: Untranslated regions. IR: Intergenic region.

Virus	GenBank Acession	Genome length (nt)	Genomic region						
			5′-UTR (nt)	ORF 1 nt/aa	IR1	ORF2 nt/aa	IR2	ORF3 nt/aa	3′-UTR (nt)
Terra Firme virus	OQ749768	8838	45	6696/2231	35	1275/425	24	669/223	94
	OQ749769	9026	45	6696/2231	35	1275/425	24	669/223	282
	OQ749770	8899	45	6696/2231	35	1275/425	24	669/223	155
	OQ749772	8834	45	6696/2231	35	1275/425	24	669/223	90
	OQ749774	9031	45	6696/2231	35	1275/425	24	669/223	287
	OQ749775	8901	45	6696/2231	35	1275/425	24	669/223	157
	OQ749777	9054	45	6696/2231	35	1275/425	24	669/223	310
	OQ749779	8885	45	6696/2231	35	1275/425	24	669/223	141
	OQ749781	9065	45	6696/2231	35	1275/425	24	669/223	321
	OQ749784	8834	45	6696/2231	35	1275/425	24	669/223	90
	OQ749786	9017	45	6696/2231	35	1275/425	24	669/223	273

Supplementary Table S3. Genomic organization of Loreto virus (LORV) strains. Virus: Identified virus name. GenBank accession: Accession number of the deposited sequence. Genome length (nt): Genome size in nucleotides. 5′-UTR (nt) and 3′-UTR (nt): Lengths of the untranslated regions. ORF1–ORF3 (nt/aa): Lengths of open reading frames in nucleotides and amino acids. IR1 and IR2 (nt): Lengths of intergenic regions.

Virus	GenBank Acession	Genome length (nt)	Genomic region						
			5′-UTR (nt)	ORF1 (nt/aa)	IR1 (nt)	ORF2 (nt/aa)	IR2 (nt)	ORF3 (nt/aa)	3′-UTR (nt)
Loreto virus	OQ749771	9133	150	7038/2345	34	1209/402	19	642/213	41
	OQ749776	9129	149	7038/2345	34	1209/402	19	642/213	38
	OQ749778	9149	149	7038/2345	34	1209/402	19	642/213	58
	OQ749780	9132	150	7038/2345	34	1209/402	19	642/213	40
	OQ749783	9148	150	7038/2345	34	1209/402	19	642/213	56
	OQ749785	9150	150	7038/2345	34	1209/402	19	642/213	58

Supplementary Table S4. Genomic organization of Wallerfield virus (WALV) strains. Virus: Identified virus name. GenBank accession: Accession number of the deposited sequence. Genome length (nt): Genome size in nucleotides. 5′-UTR (nt) and 3′-UTR (nt): Lengths of the untranslated regions. ORF1–ORF3 (nt/aa): Lengths of open reading frames in nucleotides and amino acids. IR1 and IR2 (nt): Lengths of intergenic regions.

Virus	GenBank Acession	Genome length (nt)	Genomic region						
			5′-UTR (nt)	ORF1 (nt/aa)	IR1 (nt)	ORF2 (nt/aa)	IR2 (nt)	ORF3 (nt/aa)	3′-UTR (nt)
Wallerfield virus	OQ749773	8804	57	6597/2198	29	1269/422	121	600/199	131
	OQ749782	8804	57	6597/2198	29	1269/422	121	600/199	131
